# The study protocol for a randomized controlled trial of a family-centred tobacco control program about environmental tobacco smoke (ETS) to reduce respiratory illness in Indigenous infants

**DOI:** 10.1186/1471-2458-10-114

**Published:** 2010-03-07

**Authors:** Vanessa Johnston, Natalie Walker, David P Thomas, Marewa Glover, Anne B Chang, Chris Bullen, Peter Morris, Ngiare Brown, Stephen Vander Hoorn, Ron Borland, Catherine Segan, Adrian Trenholme, Toni Mason, Debra Fenton, Kane Ellis

**Affiliations:** 1 Menzies School of Health Research, Institute of Advanced Studies, Charles Darwin University, Building 58, Royal Darwin Hospital, Darwin 0810, Australia; 2 Clinical Trials Research Unit, School of Population Health, University of Auckland, 261 Morrin Road, Glen Innes, Auckland 1072, New Zealand; 3 Centre for Tobacco Control Research, School of Population Health, University of Auckland, 261 Morrin Road, Glen Innes, Auckland 1072, New Zealand; 4 The Poche Centre for Indigenous Health, University of Sydney, Edward Ford Building A27, Sydney 2006, Australia; 5 Cancer Council Victoria, 100 Drummond Street, Melbourne 3053, Australia; 6 Centre for Health Policy, Programs and Economics, University of Melbourne, 207 Bouverie St, Melbourne 3053, Australia; 7 Kidz First and Women's Health Division, Counties Manukau District Health Board, Zealand Building 43, Western Campus Middlemore Hospital, Auckland, New Zealand; 8 Quit Victoria, 100 Drummond Street, Melbourne 3053, Australia; 9 Danila Dilba Aboriginal Health Service, 32-34 Knuckey Street, Darwin 0910, Australia

## Abstract

**Background:**

Acute respiratory illness (ARI) is the most common cause of acute presentations and hospitalisations of young Indigenous children in Australia and New Zealand (NZ). Environmental tobacco smoke (ETS) from household smoking is a significant and preventable contributor to childhood ARI. This paper describes the protocol for a study which aims to test the efficacy of a family-centred tobacco control program about ETS to improve the respiratory health of Indigenous infants in Australia and New Zealand. For the purpose of this paper 'Indigenous' refers to Australia's Aboriginal and Torres Strait Islander peoples when referring to Australian Indigenous populations. In New Zealand, the term 'Indigenous' refers to Māori.

**Methods/Design:**

This study will be a parallel, randomized, controlled trial. Participants will be Indigenous women and their infants, half of whom will be randomly allocated to an 'intervention' group, who will receive the tobacco control program over three home visits in the first three months of the infant's life and half to a control group receiving 'usual care' (i.e. they will not receive the tobacco control program). Indigenous health workers will deliver the intervention, the goal of which is to reduce or eliminate infant exposure to ETS. Data collection will occur at baseline (shortly after birth) and when the infant is four months and one year of age. The primary outcome is a doctor-diagnosed, documented case of respiratory illness in participating infants.

**Discussion:**

Interventions aimed at reducing exposure of Indigenous children to ETS have the potential for significant benefits for Indigenous communities. There is currently a dearth of evidence for the effect of tobacco control interventions to reduce children's exposure to ETS among Indigenous populations. This study will provide high-quality evidence of the efficacy of a family-centred tobacco control program on ETS to reduce respiratory illness. Outcomes of our study will be important and significant for Indigenous tobacco control in Australia and New Zealand and prevention of respiratory illness in children.

**Trial registration:**

Australian New Zealand Clinical Trials Registry (ACTRN12609000937213)

## Background

Globally, acute respiratory infections (ARI) cause more deaths and hospitalisations among Indigenous children compared with their age-matched counterparts; the greatest impact is among young children aged 0-4 years [1]. Specifically, in Australia and New Zealand (NZ), ARI is the leading cause of morbidity among Indigenous children, resulting in more hospitalisations than any other cause [2-4].

While there are multiple socioeconomic determinants of ARIs among Indigenous children, environmental tobacco smoke (ETS) exposure is arguably the most readily amenable to modification. The adverse health effects of ETS are well documented, especially its association with respiratory illness. The association between parental smoking and childhood respiratory disease is strongest at younger ages [5]. In children, exposure to ETS is causally related to the frequency and severity of respiratory illness (especially lower respiratory illness), as well as otitis media and chronic middle ear effusion [6,7]. Exposure to ETS during early childhood is also associated with an increased risk and/or worsening of pre-existing asthma symptoms among children [8,9]. Additionally, childhood ETS exposure is associated with sudden infant death syndrome (SIDS) [10].

In recognition of the damaging health effects of ETS, the World Health Organization (WHO) has prioritised the need to reduce parental smoking as a core component of improving health and development in early childhood [11]. This is especially important among socioeconomically disadvantaged populations where smoking rates among adults are higher, compared to more advantaged groups. Indigenous Australians and Māori New Zealanders are twice as likely to smoke as their non-Indigenous counterparts [12,13]. High smoking rates mean Indigenous children in these countries suffer a greater burden of ETS exposure. Australian data indicate that approximately one third of Indigenous children aged 0-14 years live in households with a regular smoker who smokes indoors compared with 9% of non-Indigenous young people [14]. While there has been a significant fall in the proportion of Maori households where smoking is permitted indoors, age-adjusted data from the 2006/7 NZ Health Survey found that Māori children and children living in neighbourhoods of high deprivation had twice the risk of exposure to ETS in their home compared to the total population of children surveyed [15].

Despite the recognition of the adverse child health effects of ETS and the scale of the problem among disadvantaged populations in particular, exposure reduction in homes is a relatively recent area of scientific study [16]. A recent systematic review of family and carer tobacco control programs for reducing children's exposure to ETS reported that of 36 studies that met inclusion criteria, 11 demonstrated a statistically significant intervention effect [17]. While the authors of this review [17] concluded that there was insufficient evidence to show which interventions were most effective, there does appear to be modest empirical support for more intensive counselling approaches for parents in this context and for interventions which focus on changing participants' attitudes and behaviours (i.e. premised on a theory of behaviour change), rather than just aiming to change knowledge alone. Notably, recent qualitative research on smoking in remote Northern Territory Indigenous communities found that Indigenous parents and carers are concerned about the health effects of ETS [18]. Moreover, their primary motivation to quit was the health of their children, in addition to positive role modelling. Among those who had failed to quit, some were already taking positive steps to minimise the exposure of their children to tobacco smoke. This reflects the importance afforded to the wellbeing of the family and in particular the fulfilment of familial responsibilities towards the care and protection of children among Indigenous Australians [19]. NZ Māori similarly have quit for the benefit of their children, with health and cost as other primary motivators to quit [20,21]. These data suggest that a family based tobacco control intervention, which was not predicated on adults quitting smoking and was focused around the welfare of children, may be particularly effective among this population.

This study aims to test the efficacy of a culturally appropriate, family-centred tobacco control program about ETS to improve the respiratory health of Indigenous infants in Australia and NZ.

### Hypothesis

Infants (<12 months) of Indigenous mothers/caregivers who receive an intensive family-centred tobacco control program about ETS, compared with 'usual care,' will have fewer health care presentations for respiratory illness.

## Methods/Design

### Objectives

The primary objective of this study is to determine the efficacy of a family-centred tobacco control program about ETS, to reduce health care presentations for respiratory illness in Indigenous infants in the first year of life. Secondary objectives include assessment of the effect of such a program on a range of measures of infant ETS exposure, including mother/caregiver's self-report of infant exposure to ETS and implementation of smoking restrictions in the home and/or car, infant urinary cotinine, household smoking status, mother/caregiver's smoking cessation and quit attempts. A process evaluation of the family-centred tobacco control program will also be undertaken.

### Study Design

**Figure 1:**
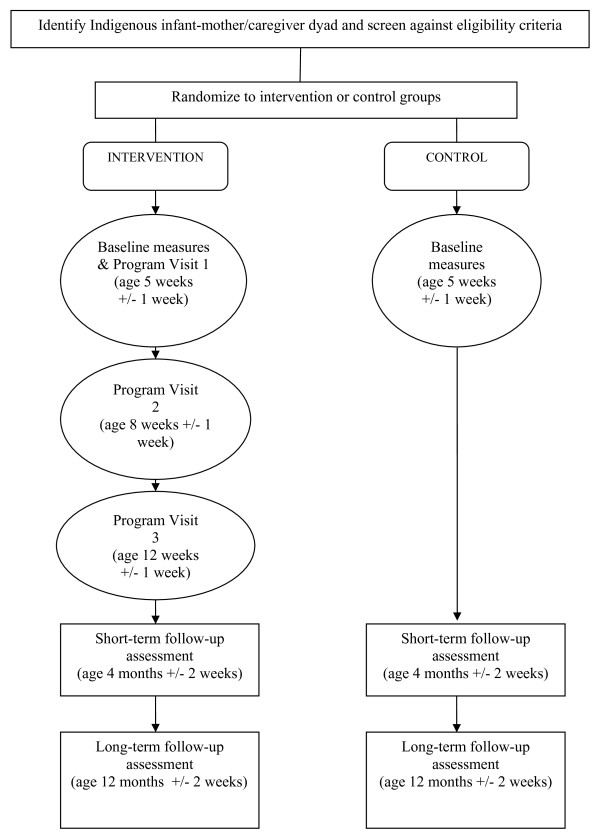
**Flowchart for a randomized controlled trial of a family tobacco control program about ETS to reduce respiratory illness in Indigenous infants**.

This study will be a parallel, randomized controlled study with allocation concealed from the study researchers. Participants will be randomly allocated to one of two study arms: the 'intervention' group who will receive the tobacco control program about ETS exposure over three home visits in the first three months of the infant's life, or the control group who will receive 'usual care' (i.e. they will not receive the tobacco control program). IHWs will be trained to deliver the intervention program, the goal of which is to reduce or eliminate exposure of infants to ETS. Data collection will occur at baseline (shortly after birth), and when the infant is four months and one year of age. The primary outcome of interest is the rate of health care presentations for doctor-diagnosed respiratory illness. Figure 1 outlines the flow of participants through the study.

### Study Population

This study will be conducted in Darwin City and the Greater Darwin area in the Northern Territory, Australia; and within the Counties Manukau District Health Board region, Manukau City, NZ. The target populations for this study are Indigenous families who reside in these two geographical areas. The sampling unit is Indigenous newborn infants and their mothers/primary caregivers (aged 16 years and over).

#### Inclusion Criteria

Infants will be eligible for inclusion if:

• They are aged between 0-5 weeks. (We will review our recruitment rate four months after the trial has commenced. If we find that we are not meeting our enrolment targets, we will consider extending our recruitment to include infants aged 5-10 weeks. This will allow us to approach mothers and their infants at 6-8 weeks when they present at community health clinics for their first immunizations.)

• Their mother/caregiver is Indigenous (defined by maternal self-identification).

• Their mother/caregiver is aged 16 years or over.

• Their mother/caregiver currently smokes or the infant lives in a household where there is at least one other person who smokes (defined as smoking at least weekly).

• Their mother/caregiver plans to reside permanently with the infant in Darwin or Greater Darwin areas of Australia or within the Counties Manukau District Health Board region, Manukau, NZ.

• Their mother/caregiver has given signed written consent to participate in this research study.

• Their mother/caregiver has given signed written consent for study staff to access the infant's health records.

• They are a singleton or the first born in a multiple pregnancy delivery.

• Their mother/caregiver speaks English and/or Māori.

#### Exclusion Criteria

Infants will be excluded from the trial if:

• They have serious neonatal respiratory complications (i.e. they require oxygen for >24 hours during their postnatal hospital stay).

• They have other serious neonatal complications (e.g. seizures, significant sepsis).

• They have major organ abnormalities (i.e. cardiac disease, congenital lung/diaphragm abnormalities, chromosomal abnormality or syndrome e.g. Down's syndrome).

The above exclusion criteria are because these infants will be given intense interventions to reduce ETS exposure regardless. Infants will also be excluded if:

• Their mother/caregiver has previously been recruited in this research study.

• They live in the same household as a mother/caregiver who has previously been recruited in this study.

### Randomisation: allocation concealment and sequence generation

Participants will be randomized by computer with stratification using permuted blocks by country (Australia, NZ) and infant age (0-5 weeks, >5-10 weeks). This will ensure a balance in these key prognostic indicators between the intervention and control groups. All participants (i.e. the infants) will be assigned a unique registration number allocated by a central computer following the submission of their details on a web-based form.

### Blinding

This is an assessor blinded trial, with only the study researchers assigning the major primary and secondary outcome measures and trial statisticians blinded to group assignment. Research assistants, who will be responsible for collecting the minor outcome measures will accompany the health workers to the participants' homes for all visits and thus cannot be blinded. The primary outcome will however be a double-blinded measure.

### Proposed intervention

Globally, Indigenous notions of health and illness are generally defined more broadly than a Western biomedical definition that focuses more often on physical health or the absence of disease [22]. In Australia, the National Aboriginal Health Strategy, [[23], p.x] defines Indigenous 'health' holistically as the physical, social, emotional, and cultural wellbeing of the individual and the community. In NZ, Te Whare Tapa Wha, a widely used Māori model of health is based on the four-sided whareuni or meeting house. It incorporates tinana (physical body), hinengaro (psychological), whānau (family and community), and wairua (spirit) [20]. Balance between these dimensions is required in order to maintain stability and good health. Poor health develops when there is a breakdown in the harmony of these four realms within the individual or in their relationship to the wider environment [20].

In this study, the intervention program about ETS will be framed around an Indigenous model of health promotion, which attends to the psychological, physical, spiritual and cultural wellbeing of the individual and the family/community, as it relates to this project. In NZ, Te Whare Tapa Wha will be used. This model has been applied to understanding Māori smoking cessation behaviour [20] and for guiding the development of culturally appropriate smoking cessation programs and strategies [24]. In Australia, the model will draw on similar concepts. The counselling/psychological component of the intervention program is founded on social cognitive theory and motivational interviewing.

Participants will be told that they will be randomly assigned to a group that receives 'usual care', or a group that receives extra home visits by an IHW, and that a variety of study measurements will be taken over a 12-month period.

• **Treatment group: **The intervention program will (i) provide information and education about the health effects of ETS exposure and use behavioural 'coaching' techniques to help mothers/caregivers and family members implement strategies to reduce the infant's ETS exposure, as well as (ii) identify the smokers among other household members and deliver culturally appropriate smoking cessation advice, counselling and treatment options as requested. An eight weeks supply of free nicotine replacement therapy (NRT) (patches or gum) will be available to participants and other household members for whom such drug therapy is indicated (i.e. they are motivated to quit, are nicotine dependent and have no contraindications to taking NRT). NRT will be provided by the IHW with appropriate counselling and follow-up. Furthermore, for those that are interested a fax referral to Quitline will be offered, with proactive call back by Quitline.

The intervention program will be delivered during three face-to-face home visits (of approximately 45-60 minutes) conducted over the first three months of the infant's life. Culturally appropriate resources (e.g. flip charts, 'No Smoking' stickers, posters, etc) will be used to assist in both education and behavioural 'coaching'. These resources will be obtained from relevant health groups in each country who hold a repository of such resources (e.g. QUIT Victoria, the Northern Territory Department of Health and Families, Auckland Regional Public Health Service). IHWs will deliver the program after appropriate training, and will complete standardised progress reports after each program session, which will be used at a weekly team meeting with the health workers and study personnel for discussion and ongoing training.

• **Control group: **The control group will receive 'usual' care through their community health provider. Usual care entails routine visits to maternal and child health providers at several defined time points during the first 12 months of the infant's life. At these postnatal visits, health providers check developmental milestones and general wellbeing. Additionally, mothers/caregivers routinely receive messages about smoking cessation and ETS exposure in their homes during these visits as part of general health promotion.

In addition to the above, the IHWs will briefly check that both groups have received the 'usual' care delivered to new mothers and their infants through routine 'well baby' visits in the first 12 months of life through their standard health provider (i.e. the study team will check these infants are not 'falling through the gaps' in the health system). This will be undertaken at baseline, when the infant is four months and one year of age. The focus will be on key health promotion messages that should have been delivered at routine community health visits (e.g. immunisations, infant nutrition/breastfeeding and safe sleeping for baby.) Mothers/caregivers will be given a few key messages if they have not received this information, e.g. clear face, sleep on back for SIDS prevention. If these visits have not been attended or if the key messages have not been received by the mothers/caregivers, they will be referred back to their usual maternal and child health provider.

### Outcome measures

Primary Outcome: *Rate of health provider presentations for new primary episodes of ARI in the first year of life*.

All participating infants will be evaluated at baseline and when the infant is four and 12 months old, for the occurrence of medically attended acute respiratory illnesses (MAARI). These are defined as new onset events, including a change from the child's baseline medical status, referable to the upper and/or lower respiratory tracts. To identify episodes of ARI mothers/caregivers will be asked at baseline, and when the infant is four and 12 months old, whether their child has had any presentation to the clinic or hospital and the names of the clinics attended. Research assistants will collect the source data relevant to the primary outcome measures, so will review the individual child's health provider and hospital clinic records (parental consent will be obtained prior to accessing records). Source documents will be photocopied, de-identified, labeled with the participant registration, and stored with the trial records. Two clinicians at each study centre will review the records and confirm documented respiratory illnesses without knowing the group allocation of the individual children. MAARI events will be evaluated by these clinicians to determine whether they are medically-attended upper respiratory infection (URI), lower respiratory infection (LRI) and/or otitis media infection (based on the definitions in Additional file 1: Table S1). Conflict will be resolved by discussion and consensus between the two clinicians. Intra-rater and inter-country reliability testing will be performed on primary outcome data from 20 participants at each site. Information on antibiotic use for the treatment of medically attended URI, LRI or otitis media events will not be collected.

Major secondary outcomes:

▪ *Rate of hospitalizations for ARI*: same case definition as primary outcome measure.

▪ *Urinary cotinine*: Urinary cotinine, a metabolite of nicotine, is recommended as the best biomarker [25]. It has a relatively long half-life (32-82 hours in children) [26]. The laboratory will use gas chromatography/mass spectrometry (GCMS) in selected-ion-monitoring mode with a stable isotope internal standard. This method provides a sensitive analytical method with high specificity [27]. Urine samples from infants will be obtained by placing several sterile cotton balls in a clean nappy. The wet cotton balls will subsequently be packed into a sterile 20 mL syringe (without needle) and the urine expressed into a 10 mL sterile specimen jar which will be delivered to the contracted medical laboratory in each country for analysis. Urinary creatinine will be measured concurrently and results expressed as the cotinine/creatinine ratio (ng/mg).

Minor secondary outcomes and likely mediators of effect:

▪ *Mother/caregiver's self-report of infant's exposure to ETS: *Number of days in the preceding seven days in which infant was exposed to ETS (same room in a house with a person who smokes, in a car with a person who smokes, or sitting outside within arm's length of someone who smokes).

▪ *Mother/caregiver's self-report of smoking restrictions in the home and car*.

▪ *Mother/caregiver's self-report of smoking cessation*: defined as mother/caregiver not smoking a single cigarette (not even a puff), in the preceding seven days. We will also be assessing prolonged abstinence (e.g. quit for 3 months at 4 month follow up; quit for 9 months at 12 month follow-up).

▪ *Mother/caregiver's self-report of number quit attempts*: defined as not smoking a cigarette for at least 24 hours.

▪ *Process evaluation indicators*: a mix of quantitative and qualitative measures to assess how well the intervention program was implemented according to protocol e.g. number of 'coaching' activities completed, obstacles and successes in delivering program, parent satisfaction with the program.

### Sample size

A total sample size of 190 provides 90% power (5% significance) to detect a 25% reduction of new episodes of respiratory illness (primary outcome) in the intervention group compared to the control group. This is based on a conservative estimate of an average of 3 health provider visits per year in the control group, compared to an average of 2.25 visits in the intervention group (assuming a Poisson distribution where the mean equals the variance). There are few published data on the community burden of ARIs in Northern Territory Indigenous children or NZ Māori children. In a recent study of disease burden and clinic attendances for young Indigenous children in two remote Northern Territory communities, the median number of presentations for upper respiratory illness in the first year of life was 7.5 (interquartile range 4-11) and for lower respiratory illness, 2.5 (interquartile range 1-5) [28]. The overall number of episodes of respiratory illness is likely to be less in an urban setting compared to that in the remote contexts [29]. Thus, it is estimated that an average of 3 visits per year be undertaken in the control group for the sample size calculation above. A maximum of 10% loss to follow-up is expected, as there are multiple contact points throughout the study for both groups and because the primary outcome (rate of respiratory infections) even in children who leave the study region before 12 months of age can be measured. Thus, 210 children in each country will be recruited. Given that there are limited data to base the likely effect size on, it is possible that the effect size chosen may be optimistic. By combining data from NZ and Australia the sample size is increased to 380 participants (420 if loss to follow-up is factored in), which will provide 90% power (at 5% significance level) to detect an 18% reduction in new episodes of ARI in the intervention group compared to the control group.

### Recruitment strategies

In Australia and NZ, mothers/caregivers will be approached for recruitment into the study through a range of community, Indigenous-controlled health and government hospital services. We will approach both pregnant women and new mothers to ascertain their interest in participating in the trial. If pregnant women are interested in the study, we will re-approach them after they have given birth. Eligible and interested mothers will be asked for their consent for randomization. Documented written consent will be obtained from all participants prior to entering the study. A range of project-specific advertising material will be produced (e.g. clinic posters, brochures) and local Indigenous media may be used to bolster recruitment.

### Planned recruitment rate and risk of loss to follow up

In 2006, there were approximately 240 Indigenous infants born to mothers who were resident in Darwin, Australia. We anticipate that 90% or 216 Indigenous infants/mothers will be eligible for recruitment annually into this study. In a separate study based in Darwin to improve ear health outcomes, 55% of Indigenous women approached in pregnancy have consented to participate in a randomized controlled vaccine study (pers. comm., Dr Andrews [Menzies School of Health Research], 10 January 2008). Using these figures, we could confidently anticipate that approximately 108 eligible Indigenous mothers will agree to participate in our study each year, or 9 per month. This is likely to be an underestimation, as our proposed study is less invasive than the ongoing vaccine trial (which requires immunisation and repeated blood samples).

It is anticipated that recruitment will be faster at the Auckland site owing to a larger eligible population. Approximately 580 babies are born each month at Middlemore Hospital, of which 21% are Māori [pers. comm., N. Knetsch, Counties Manukau District Health Board]. Data reported in 2003 showed that about 55% of Māori pregnant women in NZ were smoking at the time of conception, and 15% of these women quit smoking during their pregnancy[30] From these data we can estimate that at least 57 Māori new mothers that smoke will be available per month to approach about the trial (mothers that are non-smokers but live in households with other smokers are also eligible). We anticipate that 90% or 51 infants (assuming no twins) will meet the inclusion criteria. We estimate that about 60% of women approached will agree to participate (i.e. 31 per month). Consequently it is expected to take about seven months to recruit the 210 people required for the NZ arm of the trial.

**Figure 2:**
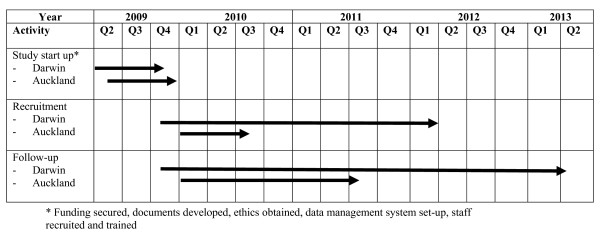
**Timeline for a randomized controlled trial of a family tobacco control program about ETS to reduce respiratory illness in Indigenous infants**.

Figure 2 outlines the timeline for this study at both sites.

### Withdrawal criteria

Participants may be withdrawn from the study if one or more of the following occurs:

• Voluntary withdrawal: A parent or primary caregiver can voluntarily withdraw their infant from the project at any time without having to provide a reason for doing so.

• Failure to meet eligibility criteria: An infant will be withdrawn from the project if their mother/caregiver has previously signed a consent form but they do not meet all the eligibility criteria at the baseline visit (when the infant is five weeks old +/- one week).

• Failure to be located after multiple follow-up home visits

• Death of infant

• Significant illness requiring prolonged hospitalization

• A serious and irreconcilable protocol violation (as determined by the Investigators)

In the event that a study participant dies, their family contact will be asked if they require the information recorded to be disposed of as per other participants at the completion of the study, or whether they wish the information to be returned to the family.

### Data Management

The design and management of all databases associated with this trial will be undertaken by the data management and information technology groups at the Clinical Trials Research Unit (CTRU), University of Auckland. The databases will be constructed in Oracle. Validation rules for each Case Report Form (CRF) will be specified by the site study managers, in association with the CTRU data manager. These rules will include range checks so that inaccuracies in data collection can be identified early. A query will be raised as soon as any values are entered that are outside the allowed range or if data are missing. The research assistants at each site will amend the CRF's as soon as a query is raised. All information collected from participants will be treated as strictly confidential. De-identified data will be stored in computers under a password secure file. Paper records will be stored under numerical code in a locked filing cabinet within a secure office area, and will only be accesses by approved study staff.

### Data monitoring

An independent person will be appointed to monitor the study conduct. This monitor will audit both Australia and NZ study sites and the CTRU during the trial to ensure that the study protocol is being adhered to.

At the study sites the monitor will audit every randomized participant's records to ensure their existence, that they meet the inclusion criteria and have provided written and signed informed consent, and that the NRT is been distributed within the limits of the protocol. The monitor will review the study documentation and records held at each site to ensure that (1) documentation is up-to-date [i.e. correct version of protocol and Manual of Procedures] and (2) record keeping meets the requirements specified in the protocol and complies with regulatory requirements. The monitor will visit each site early on during the study (after ten participants have been randomized), at study close-out and once during the course of the trial. At least 10% of paper copies of the CRF's will be checked for consistency with the electronic records by the monitor. 100% of electronic ARI endpoint data will be checked against source data.

The monitor will audit the sites which hold the NRT to check that NRT supply records are in order and that there are sufficient supplies remaining, that the NRT are being stored appropriately and are not being used beyond expiry dates, and that the handling of unused NRT complies with study procedures.

A Data Safety and Monitoring Committee will not be established as the trial does not meet any of the criteria stipulated by Ellenburg et al. (2002) for setting up such a Committee [31].

### Data Analyses

Statistical analyses will be performed by statisticians at the CTRU. The statistical unit at Menzies School of Health Research (Menzies) will play an advisory role. Data from the trial will be entered into an Oracle database at the CTRU, and then extracted into SAS version 9.2 (SAS Institute Inc. Cary NC), and R version 2.8.1 (R Foundations for Statistical Computing) for analysis. Data analyses will be specified *a priori *in a statistical analysis plan prepared by the study statistician (and agreed upon by all members of both the NZ and the Australian Steering Committees). No planned interim analyses will be undertaken of the outcome data. A baseline data paper will be prepared after all baseline data for both countries has been collected. The number of episodes of ARI experienced after the intervention for each group will be analysed on an "intention to treat" basis. Each of the primary and secondary outcome variables will be examined separately. There are no plans to combine outcome variables into composite variables. Data will be analysed for each country separately and combined. All ETS exposure variables will be treated and examined as outcome variables, rather than as co-variates of the primary outcome variable. Intra-rater and inter-country agreement for grading of the primary outcome assessment will be assessed using the Kappa statistic (unweighted), with 95% confidence intervals for the kappa statistic calculated using the method described by Altman [32].

#### Baseline characteristics

All known potential confounders will be measured at baseline, including mother/caregiver's smoking status (and stage of change), education level, marital status, breastfeeding status, smoking status of partner and other household members, crowding, and exposure of infant to other sources of environmental smoke (e.g. cannabis, open fires). Thus, comparisons of the intervention and control groups will be performed both unadjusted and adjusted for these known confounders. This second adjusted analysis will control for any maldistribution after randomisation of the confounders between the two groups.

#### Treatment effects

Analysis of the primary outcome will involve comparing the rate of respiratory illness between the two groups. Simple unadjusted rates, relative risks and 95% confidence intervals will be obtained in the first instance, with subsequent multiple regression analysis adjusting for other variables. Two forms of regression analysis will be considered for the primary outcome: poisson regression analysis and negative binomial if there is evidence of overdispersion or underdispersion. Analysis of secondary outcomes will be conducted using standard statistical procedures applicable to categorical or continuous data. A per-protocol analysis will also be performed in order to check the robustness of the results. All tests of significance will be two-tailed.

#### Procedures to account for missing data

For treatment effects, sensitivity analyses will be carried out to determine the effect of missing data.

### Ethics

The study will be conducted in accordance with the Australian National Health and Medical Research Council Guidelines on 'Ethical Matters in Aboriginal and Torres Strait Islander Health Research in Australia' [33]. In Australia, the Child Health Indigenous Reference Group at Menzies will monitor the study's progress. It includes representatives from consumer organizations (e.g. local Indigenous controlled health services). This group meets on a quarterly basis and the project team will provide reports to this committee describing the progress, outputs and outcomes of the project. The Reference Group will need to authorise any publication or dissemination of the research findings. Ethics approval in Australia has been granted by the Human Research Ethics Committee at Menzies (Ethics Number 09/32). Ethics approval in NZ has been granted from the Northern Region Human Ethics Committee (Ethics Number NTY/09/09/091). The Counties Manakau District Health Board Research Committee, Counties Manakau District Health Board Māori Research Review Committee and the CTRU's Māori Research Advisory Committee have given support and input to this study and will continue to provide guidance as it progresses. Participants and communities will be acknowledged at publication or presentation of the results.

### Trial management

Project Managers will be responsible for the day to day management of the trial, one in Darwin and the other in Auckland. The Project Managers' responsibilities include the development of Standard Operating Procedures, maintaining an up-to-date collection of essential documents (in line with GCP requirements), monitoring recruitment rates, attending to participant queries or concerns and managing the fieldwork staff.

Two IHWs in each country will be responsible for recruiting participants into the study and for delivering the 'intervention' program, as well as collecting process evaluation data. The development of Indigenous research workforce capacity is a vital aspect of this trial. Research assistants will collect urine samples and administer the face-to-face questionnaire to participants. They will also be responsible for data entry and data cleaning, and together with the IHWs, ensure that participant follow-up appointments are completed during the scheduled window periods.

Two clinical investigators in Darwin and Auckland will review participating infants' clinical notes and code the clinical outcome data.

## Discussion

Exposure to ETS is a strong but potentially preventable contributor to respiratory illness among young Indigenous children, when household ETS exposure of children is at its peak. Interventions aimed at encouraging smoking cessation as well as reducing exposure of Indigenous children to ETS have the potential for significant benefits for Indigenous communities. This community-based, international trial has been designed to provide high-quality evidence of the efficacy of a theoretically and culturally sound, intensive family-centred tobacco control program to reduce ETS exposure among Indigenous peoples and so reduce the burden of ARI. As such, it will make a valuable contribution to future updates of the Cochrane review of family and carer smoking control programmes for reducing children's exposure to environmental tobacco smoke. The inclusion of a process evaluation as part of the study will inform the progress and shed additional light on the outcomes of the trial. Finally, this study seeks to incorporate Indigenous models of health to inform the design of the intervention and recruitment methods, and emphasizes Indigenous capacity building at all levels.

## List of abbreviations

**ARI: **Acute respiratory illness; **ETS: **Environmental tobacco smoke; sometimes referred to as second hand smoke. Usually refers to cigarette smoke in the environment of people who do not smoke; **CTRU: **Clinical Trials Research Unit; Auckland, NZ; **IHW: **Indigenous health worker - either Aboriginal or Torres Strait Islander or Māori; **Menzies: **Menzies School of Health Research, Darwin, Australia; **NRT: **Nicotine replacement therapy, available in a range of forms such as patch, gum lozenges, tablets and nasal spray. In this research study, NRT will be dispensed in the form of nicotine patches and/or gum only.

## Competing interests

The authors declare that they have no competing interests.

## Authors' contributions

VJ is an Australian Investigator. She contributed to the study design and drafted the first version of the study protocol. She has led the development of the intervention program, the questionnaires and the study set-up in Australia. NW is the Principal Investigator for this trial in NZ. She contributed to developing and writing the final version of the study protocol. She has contributed to the development of the study questionnaires and has led the study set-up in NZ. DT is the Principal Investigator for the trial in Australia. He contributed to the study design and drafting of the study protocol. MG is a Māori health researcher in NZ, and a co-investigator on this study. She was instrumental in developing the culturally appropriate intervention program and contributed to the development of the final study protocol. She led the consultative process with Māori and assisted with the recruitment and training of the Māori IHWs and research assistants. AC is an Australian Investigator. She originally conceived of the study and contributed to the study design. CB is a NZ Investigator and contributed to developing and writing the final version of the study protocol, especially in relation to the definition of the primary outcome. PM is an Australia Investigator. He contributed to the conception and design of the study. NB is an Aboriginal health researcher and an Australian Co-investigator. She contributed to the study design, particularly in relation to developing a culturally appropriate intervention and in providing advice on recruitment and retention of Indigenous families in Darwin. SV is a NZ Investigator. He is the trial statistician, and contributed to the protocol in matters relating to randomisation, study power and statistical analyses. RB is an Australian Investigator and contributed to the development of the questionnaire to assess the secondary outcomes for the study. CS is an Australian Investigator and contributed to the conception of the study. She also provided expert input initially into the questionnaire design. KE is an Australian Investigator and contributed to the development of the intervention program and provided advice on recruitment and retention of Indigenous families in Darwin. TM is an Australian Investigator and contributed to the development of the intervention program and training of the Australian IHWs. DF is a NZ Investigator. She contributed to the study protocol, the development of the intervention program, and advice on recruitment and retention of Indigenous families in South Auckland. KE is an Australian Investigator and contributed to the development of the intervention program and provided advice on recruitment and retention of Indigenous families in Darwin.  All authors have provided critical review of this manuscript and have approved the final protocol.

## Pre-publication history

The pre-publication history for this paper can be accessed here:

http://www.biomedcentral.com/1471-2458/10/114/prepub

## Supplementary Material

Additional file 1 **Case Definitions for Acute Respiratory Infection**. A table providing full definitions of acute respiratory infection for this study Click here for file
