# Supplementary material for: The study protocol for a randomized controlled trial of a family-centred tobacco control program about environmental tobacco smoke (ETS) to reduce respiratory illness in Indigenous infants
Source: BMC Public Health. 2010 Mar 7;10:114. doi: 10.1186/1471-2458-10-114 (PMC2846866; doi:10.1186/1471-2458-10-114)
Supplement: Additional file 1 — Case Definitions for Acute Respiratory Infection. A table providing full definitions of acute respiratory infection for this study [file 1471-2458-10-114-S1.DOC]

Table 1: Case Definitions for Acute Respiratory Infection

| **Definition of an episode of upper respiratory infection (URI)**  An URI event is one that has a medical diagnosis of upper respiratory tract infection (URTI) or signs and symptoms suggestive of URTI, with the following terms used by the medical care provider:  • Runny nose  • Blocked nose  • Dry cough  • Sore throat  • Cold  • Influenza |
| --- |
| **Definition of an episode of lower respiratory infection (LRI)**  A LRI event is one that has a medical diagnosis of bronchiolitis, croup (laryngotracheobronchitis), bronchitis or pneumonia. In the absence of such a medical diagnosis, the occurrence of LRI events will be determined by two clinicians after review of the medical records and based on the presence of cough, retractions, rhonchi, wheezing, crackles or rales, with or without symptoms (by history or clinical findings) of coryza, fever or apnea. Treatment consistent with a diagnosis of infection (e.g. antibiotics) may also be used to determine diagnosis in the presence of appropriate symptom description. |
| **Definition of an otitis media episode**  Otitis media will be recorded as the diagnosis if the following terms are used by the medical care provider:  • acute otitis media  • acute tympanic membrane perforation  • bulging tympanic membrane  • red tympanic membrane with fever  • otitis media with effusion  • middle ear effusion  A new episode will be defined as a physician-diagnosed otitis media in either ear after a normal middle ear exam of the ear in question or an episode of acute otitis media ≥ 21 days after the previous episode. A diagnosis of persistent middle ear effusion/perforation will NOT be recorded as a new otitis media event |
